# Lymph node invasion by tumor cells modifies the distribution of dendritic cell subsets and memory T cell profiles in human cancer patients

**DOI:** 10.1186/2051-1426-2-S3-P31

**Published:** 2014-11-06

**Authors:** Nicolás Gonzalo Núñez, Ana Tereza Nadan, Louis Pérol, Maud Milder, Sophie Viel, Philippe De La Rochere Philippe, Delphine Loirat, Sastre-Garau Xavier, Christine Sedlik, Sebastian Amigorena, Eliane Piaggio

**Affiliations:** 1 Institut Curie, Paris, France

## Introduction

In human breast cancer, the invasion of tumor-draining lymph nodes (TDLNs) is an important step in disease progression and has predictive value [1]. TDLN dendritic cells (DCs), which are comprised of lymphoid-organ-resident and skin-derived migratory DCs, present tumor antigens to the naïve T cells and induce their activation and polarization into different functional subsets (Th1, Th2, Th17, Th22, Tfh, regulatory T cells) that will lead to antitumor T cell responses or to tolerance [2].

## Objective

Systematic comparison of the immune profile of Invaded (INV) versus Non-invaded (NI) TDLNs would help to identify those immunomodulatory mechanisms associated to the presence of the tumor that could condition the response to immunotherapy.

## Material and methods

TDLNs from 70 untreated breast cancer patients undergoing surgery at Institut Curie Hospital were obtained in accordance with institutional ethical guidelines. Samples were analyzed by multi-color flow cytometry. For statistical analysis, Wilcoxon matched paired test or Mann-Whitney test was performed using Prism (GraphPadSoftware)

## Results

We studied the distribution of 6 different DC subpopulations and observed in INV TDLNs a significant decrease in the percentage of BDCA1+ DCs (*P*<0.05) and a significant increase in the percentage of CD11c+HLADR+CD14+cells (*P*<0.01), including macrophages and inflammatory DCs, compared to NI TDLNs (*P*<0.05). We also found a significant lower frequency of naïve conventional and regulatory T cells in INV TDLNs (*P*<0.05). Both, in NI and INV TDLNs, memory conventional and regulatory T cells were highly polarized, mainly to the Th1 phenotype, but also to the Th2, Th17, Tfh and Th22 phenotypes, as determined by the expression of a panel of chemokine receptors and transcription factors. Notably, in INV TDLNs, a significantly higher proportion of regulatory and conventional T cells were Th1-polarized (*P*<0.05). Further functional analysis showed that after ex-vivo PMA/Iono stimulation, the Th1-polarized conventional T cells, but not the Th1-polarized regulatory T cells produced high amounts of IFN-γ, being the IFN-γ production significantly higher in INV TDLNs (*P*<0.05).

## Conclusion

Overall, we observed that immune cells from metastatic TDLNs show evidence of high activation (increased proportion of inflammatory DCs and of Th1-polarized memory T cells) highlighting their potential role in the anti-tumor immune response.
